# Feasibility of Leveraging Consumer Wearable Devices with Data Platform Integration for Patient Vital Monitoring in Low-Resource Settings

**DOI:** 10.1155/2024/8906413

**Published:** 2024-02-08

**Authors:** Elsie Effah Kaufmann, Richmond Tackie, J. Benjamin Pitt, Samuel Mba, Bismark Akwetey, Danielle Quaye, Godfrey Mills, Clement Nyame, Henry Bulley, Matthew Glucksberg, Hassan Ghomrawi, William Appeadu-Mensah, Fizan Abdullah

**Affiliations:** ^1^Department of Biomedical Engineering, University of Ghana, Accra, Ghana; ^2^Northwestern University Feinberg School of Medicine, Chicago, USA; ^3^Department of Computer Engineering, University of Ghana, Accra, Ghana; ^4^Korle Bu Teaching Hospital, Accra, Ghana

## Abstract

Manual monitoring of vital signs, which often fails to capture the onset of deterioration, is the main monitoring modality in most Ghanaian hospitals due to the high cost and inadequate supply of patient bedside monitors. Consumer wearable devices (CWDs) are emerging, relatively low-cost technologies for continuous monitoring of physiological status; however, their validity has not been established in low-resource clinical settings. We aimed to (1) investigate the validity of the heart rate (HR) and oxygen saturation (SpO2) data from two widely used CWDs, the Fitbit Versa 2 and Xiaomi Mi Smart Band 6, against gold standard bedside monitors in one Ghanaian hospital and (2) develop a web application to capture and display CWD data in a clinician-friendly way. A healthy volunteer simultaneously wore both CWDs and blood pressure cuffs to measure HR and SpO2. To test for concordance, we conducted the Bland-Altman and mean absolute percentage error analyses. We also developed a web application that retrieves and displays CWD data in near real time as text and graphical trends. Compared to gold standards (patient monitor and manual), the Fitbit Versa 2 had 96.87% and 96.67% measurement accuracies for HR, and the Xiaomi Mi Smart Band 6 had 94.24% and 93.21% measurement accuracies for HR. The Xiaomi Mi Smart Band 6 had 98.79% measurement accuracy for SpO2. The strong concordance between CWD and gold standards supports the potential implementation of these devices as a novel method of vital sign monitoring to replace manual monitoring, thus saving costs and improving patient outcomes. Further studies are needed for confirmation.

## 1. Introduction

There are five traditional vital signs that are considered the gold standard for monitoring physiologic status: respiratory rate (RR), heart rate (HR), body temperature, blood pressure (BP), and blood oxygen saturation (SpO2). As such, there is a need for continuous monitoring of these vital signs in hospitalized patients [1]. Although changes in vital signs accurately predict clinical deterioration leading to adverse events, these changes often go unnoticed in low- and middle-income countries (LMICs) or are detected late [2–4]. This is due to the inability to frequently monitor and interpret vital signs, inadequate recording of vital signs, or because of an inappropriate response to abnormal values [3, 5–7]. The current bedside vital sign monitors provide much needed continuous vital sign data; however, several factors make these monitors unsustainable and ineffective in resource-limited settings [8]. The bedside monitors are expensive, and their service and maintenance requirements generally far exceed local resources. Together with voltage instability, these factors contribute to the short lifespan of conventional monitoring equipment.

Additionally, human resource factors make conventional monitoring devices less than ideal due to the large amount of time required to take patient vitals and record them manually at the appropriate frequency. The two major contributing factors to this phenomenon are a lack of monitoring equipment and a shortage of staff to monitor vital signs [9, 10]. In 2017, a survey of nurse anesthetists trained in Ghana showed that 29% work alone or in pairs and only 60% have access to capnography and less than 80% have EKG monitoring [11]. In a recent study of 232 nurses in Ghana, 44% of nurses reported adequate staffing and resources [12]. A quality improvement initiative at a regional hospital in Ghana that included improvements in staffing and equipment was able to cut post-C-section mortality due to hemorrhage from 14.8% to 1.6% over 4 years [13].

Wearable technology encompasses a myriad of devices either worn directly or loosely attached to a person [14]. Consumer wearable devices (CWDs) are emerging and increasingly ubiquitous wearable technology that enhance the continuous monitoring of human physiological data during daily activities or in a clinical environment with the benefit of lessening discomfort and/or interference with normal human activities [15], the use of which has increased among both adults and children [16]. CWDs have incorporated sensors that are comparable to clinical-grade bedside monitors and can transmit data in near real time to a smartphone using Bluetooth technology and then to a cloud computing service that can be shared with a clinical team [17]. With these capabilities, CWDs such as the Fitbit and the Xiaomi have great potential as a wireless monitoring system alternative to traditional methods of monitoring.

CWDs have been implemented in some clinical settings as a supplement for vital sign monitoring. In the United States, the ViSi Mobile device from Sotera Wireless [18] is a commercial product that has been accepted by the Food and Drug Administration (FDA) as a health device and is being used as a monitoring device in some hospitals. It reads and records SpO2 levels, skin temperature, electrocardiogram (ECG), HR, BP, and RR [19]. In addition, SensiumVitals from Sensium Healthcare Limited [20] in the UK is an FDA-approved and Conformité Européenne-marked wearable vital sign monitoring system that records HR, RR, and body temperature [21].

We believe that Bluetooth-based wearable physiology monitors, such as Fitbit or Xiaomi, connected to central monitoring stations may be able to overcome the limitations of currently used vital sign monitors and improve the care of patients. The aim of this study was to investigate the validity of the HR and SpO2 data from two widely used CWDs, the Fitbit Versa 2 and Xiaomi Mi Smart Band 6, against gold standard clinical bedside monitors used in one Ghanaian hospital, to develop a fully functioning web application to capture and display CWD data in a clinician-friendly way and assess the feasibility of incorporating this platform into clinical practice.

## 2. Materials and Methods

### 2.1. Participants

A healthy 22-year-old male volunteer with no medical or surgical history was used as the study subject. This study formed part of a larger study for which ethical clearance was obtained (KBTH-IRB 00092/2022).

### 2.2. Devices Used in the Study

The Fitbit Versa 2 and Xiaomi Mi Smart Band 6 were selected for the study to assess the feasibility of utilizing them as alternatives and/or replacements for the manual mode of vital sign measurement in various hospitals in Ghana. These devices were chosen based on the success of previous studies using similar devices for the monitoring of physiologic data [22–27]. The Philips MX450 patient monitor and a portable BP and HR monitoring device were used for the gold standard measurements. Both wearable devices were worn on the wrist of the volunteer's left arm, and 5 ECG leads were placed on the chest of the volunteer and connected to the Philips monitor for HR data.

### 2.3. Study Procedure

The study was carried out in a closed room environment at the University of Ghana Medical Center (UGMC) over a two-day period. The study was conducted in an environment with a temperature of 25 degrees Celsius. The two devices were placed on the dominant arm of the volunteer and synchronized with their respective mobile applications on a smartphone.

Recording of vital signs commenced at least one minute after placement of the ECG electrodes. The volunteer was laid on a table in a supine position to ensure accurate HR recordings as previously done in a similar study [28]. The HR and SpO2 data from both the Philips MX450 patient monitor and the CWDs were recorded in intervals of one minute.

### 2.4. Data Collection and Processing

The Philips MX450 patient monitor was set to record HR and SpO2 data every minute. To obtain SpO2 data, a pulse oximeter was placed on the finger of the volunteer and connected to the Philips patient monitor. One member of the team had the task of recording the HR and SpO2 data from the Philips MX450 patient monitor into a comma-separated values (CSV) file. The data retrieved from the patient monitor came with the time at which it was recorded and data from each CWD were matched based on time stamps. A total of 116 HR data points were recorded by the patient monitor on day one and 121 HR data points on day two, summing up to a total of 237 HR data points obtained from the Philips MX450 patient monitor. The Philips MX450 patient monitor recorded 116 SpO2 data points on day one and 121 on day two, also summing up to a total of 237 SpO2 data points.

To retrieve data recorded by Fitbit, an application programming interface (API) was registered on the Fitbit website and then a python code was written to make a get request to the registered API with Fitbit. The data was retrieved in a CSV file format with time stamps and in intervals of one minute. The retrieved CSV file from Fitbit contained 135 HR data points for day one and 121 for day two, summing up to a total of 256 HR data points.

To retrieve data from Xiaomi, an account was registered with a third-party website, Huami. Data recorded on the Xiaomi device were retrieved in a CSV file format via the Huami website. The data recorded by the Xiaomi device contained 130 HR data points on day one and 121 on day two making a total of 251 HR data points. Data retrieved from the Xiaomi device were also recorded every minute and came with the time of recording as well. The Xiaomi device also recorded SpO2 with 36 data points on day one, 115 data points on day two, and 151 SpO2 overall data points. The SpO2 data were recorded manually and processed into a CSV file. HR data obtained from all the devices were processed into a single CSV file for each day and combined to form an overall HR dataset for each device in a single CSV file. This resulted in a total of three CSV files for day one, day two, and overall. The data from all the devices were matched using the time at which they were recorded. A total of 116 data points were processed into a single CSV file for each device on day one, and 121 HR data points were processed for day two, resulting in 237 data points for HR. Thirty-six SpO2 data points were processed into a CSV file for Philips MX450 patient monitor and Xiaomi on day one, 115 SpO2 data points on day two, and an overall of 151 data points for SpO2.

To obtain manual data from the portable BP monitor, the cuff was worn on the left arm around the biceps and triceps 2-3 cm from the elbow and fastened tightly. The cuff was connected to the BP device, which provided pressure on the arm for the physiological data to be measured. HR data was recorded in intervals of 30 minutes and then later in intervals of one minute to obtain sufficient data points. The time at which the data was taken was then recorded. A total of 116 HR data points were obtained.

### 2.5. Statistical Analysis

All statistical analyses on data collected and processed were performed in Python (Jupyter Notebook) which was locally installed on a computer. The Python libraries used were NumPy, SciPy, Matplotlib, seaborn, statsmodels, and pandas.

The normality of the data collected was tested to identify whether the dataset followed a Gaussian distribution in order to select the most appropriate tests for the dataset [29]. To achieve this, quantile-quantile (Q-Q) plots, histogram plots, and the Shapiro-Wilk and Kolmogorov-Smirnov tests were used. All tests showed that the datasets did not follow a normal distribution and therefore only statistical analyses valid for nonnormally distributed datasets were used to evaluate the performance of the CWDs against the patient monitor and the manual monitoring device.

Three major analyses were performed on the datasets in Python. The first was correlation analysis. The Spearman correlation coefficient was calculated in Python using the in-built libraries. The Spearman correlation coefficient measures the relationship between two variables and in this case two different methods of measurement [30, 31]. The import of the Spearman correlation analysis was to determine whether the measurements from the Fitbit and Xiaomi devices were significantly different (lower or higher) compared to values recorded by the gold standard devices. The Bland-Altman analysis was also performed to compare measurements made with the various devices. The Bland-Altman analysis is a plot that shows the level of agreement between a new method of measurement and an already existing method of measurement by estimating the mean difference and the level of agreement between the two methods used in measuring the same quantity over a 95% confidence interval [32]. The plot also gives information on whether the new method overestimates or underestimates the measurement. Additionally, the Bland-Altman plot identifies possible outliers, making it suitable for the study. The final metric that was evaluated was the mean absolute percentage error (MAPE) to assess the accuracy of the CWDs. The MAPE gives errors in estimating a quantity by a new method of measurement relative to a gold standard method in percentage [33].

For further correlation analyses, the Mann–Whitney *U* test was used to compare the differences between the heart rate data from the patient monitor and the two wearable devices. This nonparametric test was used to assess the relationship between the gold standards and the wearable devices using the null hypothesis Ho: there exists a significant relationship between the gold standards and the wearable devices in terms of heart rate measurement. This analysis was also performed in Python.

### 2.6. Web Application Development

To develop a web application to store and display data collected from the wearable devices, the concept of the application (UI/UX) was designed in Figma. The design was translated into a front-end design using hypertext markup language (HTML), cascading style sheets (CSS), and JavaScript programming languages. The backend of the web application was developed in Django.

**Figure 1:**
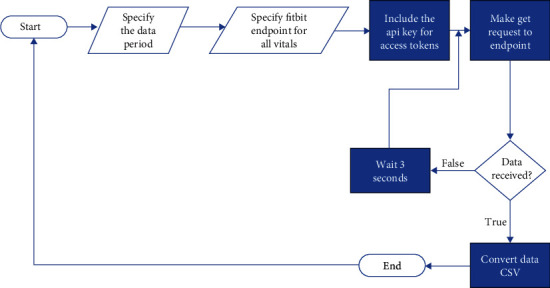
Flow chart showing how to make a get request from the Fitbit API.

**Figure 2:**
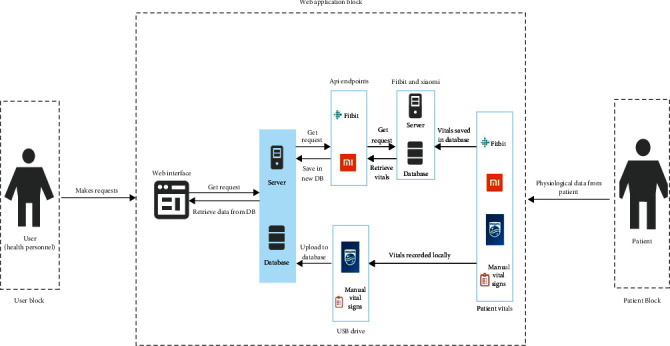
Architectural diagram of the system showing various functions, their implementations, and interactions with each other.

The backend was designed to communicate with the CWD web application programming interface (API) to retrieve an individual's HR (Figure 1). With this design, users will be able to view patients' HR and SpO2 data (Figure 2). The SQLite 3 database tables were successfully converted to Heroku's Postgres database. The architectural diagram of the web application is described as follows:

When a user submits a request, such as uploading manual vitals, viewing patient data, and searching for a patient, the web server is able to interact with the database, retrieving or posting the data requested. Essentially, the following actions may be executed:

1. Display of patient data on the web interface for the user to visualize
2. Fitbit and Xiaomi APIs communicate with their web servers to retrieve physiological data when the user makes a request to visualize patient heart rate and pulse oxygenation data
3. Patients are registered on the Fitbit and Zepp Life apps using an email and password with the wearables worn on the wrist
4. Physiological data obtained from patients is stored on cloud databases via the android application web servers
5. Physiological data is synced every 15 minutes to save data into the cloud databases
6. Data obtained from manual monitoring is transcribed into an Excel sheet and saved as CSV
7. Data obtained from the Philips IntelliVue MX450 patient monitor is recorded with a USB drive and saved as a CSV file
8. Data obtained from both the manual monitoring and the Philips patient monitor can be uploaded into the web application's database

## 3. Results

### 3.1. Statistical Analysis

Normality tests conducted on the datasets using the Shapiro-Wilk and Kolmogorov-Smirnov tests demonstrated that all the datasets were not normally distributed as the recorded *p* value in each case was less than 0.05.

**Figure 3:**
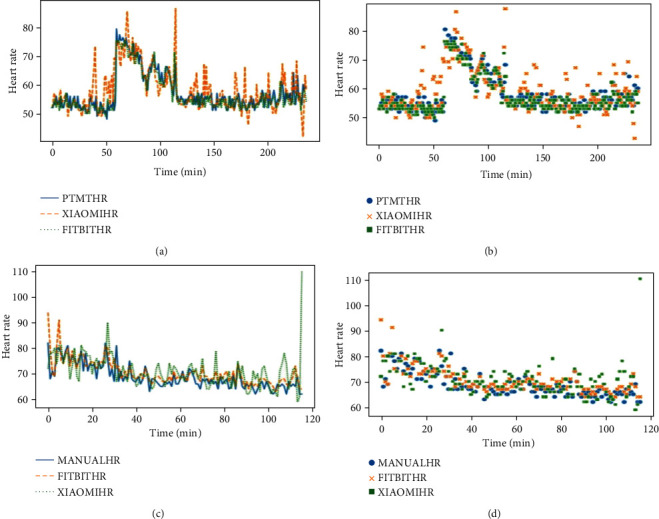
Line and scatter plots of heart rate measurements obtained from Xiaomi band (XIAOMIHR) and Fitbit (FITBITHR) compared to the gold standards of Philips IntelliVue MX450 patient monitor (PTMTHR) and manual blood pressure monitor (MANUALHR). (a) Line plot of PTMTHR, XIAOMIHR, and FITBITHR measurements. (b) The same information in a scatter plot. (c) Line plot of MANUALHR, XIAOMIHR, and FITBIT HR measurements. (d) The same information in a scatter plot.

The comparison between the wearable devices and the gold standard measurements shows a positive correlation for the evaluated Spearman correlation coefficient (Table 1 and Figure 3). Fitbit recorded a strong positive correlation of 0.749730 for HR against the patient monitor, which is a very good linear relationship as expected. Xiaomi also recorded a strong positive correlation of 0.607200 for HR against the patient monitor. Xiaomi and Fitbit recorded a correlation coefficient of 0.422363 and 0.841943 for HR, respectively, when compared to the manual mode of measurement.

**Figure 4:**
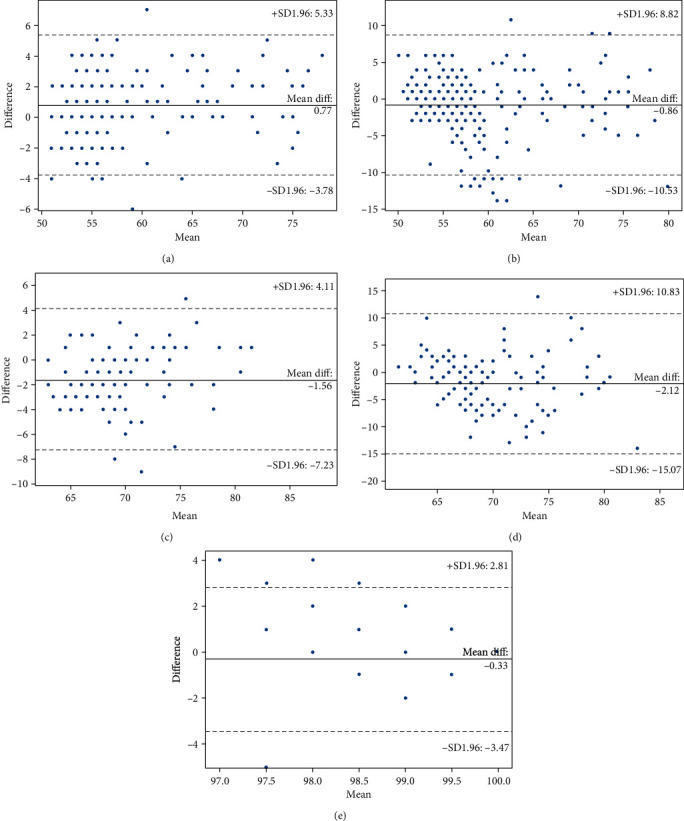
Bland-Altman plots of CWDs against the gold standards. (a) Fitbit heart rate against the Philips IntelliVue MX450 patient monitor heart rate measurement. (b) Xiaomi heart rate against the Philips IntelliVue MX450 patient monitor heart rate. (c) Fitbit heart rate against the manual BP and HR smart monitor heart rate measurement. (d) Xiaomi heart rate against the manual BP and HR smart monitor heart rate measurement. (e) Xiaomi SpO2 against the Philips IntelliVue MX450 patient monitor SpO2 measurement.

The Bland-Altman plot for Fitbit HR against patient monitor (Table 2) showed a mean difference of 0.63 bpm, 0.91 bpm, and 0.77 bpm on day one, day two, and overall, respectively (Figure 4). This means that the Fitbit device was overestimating the HR by 0.63 beats per minute on day one, 0.91 beats per minute on day two, and an overall overestimation of 0.77 beats per minute. The Bland-Altman plot for Xiaomi against the patient monitor showed a mean difference of -1.22 bpm on day one, -0.51 bpm on day two, and an overall mean difference of -0.88 bpm. This result means that Xiaomi underestimated the HR measurement by 1.22 bpm on day one, 0.51 bpm on day two, and an overall underestimation of 0.88 bpm. The Bland-Altman plot for Xiaomi also revealed quite a large limit of agreement between -10.53 and 8.82 compared to that of Fitbit. Only six and fifteen outliers were found in the plot for Fitbit and Xiaomi, respectively, out of the 237 data points which means that the remaining data points lie in the 95% confidence interval (Figures 4(a) and 4(b)).

The Bland-Altman plot for Fitbit HR data against the manual monitoring device showed that Fitbit was underestimating HR measurement by 1.56 beats per minute with a limit of agreement between -7.23 and 4.11 with three outliers (Figure 4(c)). When the results from Fitbit against the patient monitor were compared to Fitbit against the manual monitor, it can be observed that the limit of agreement of Fitbit against the manual monitor is larger than that against the patient monitor; this indicates that Fitbit agrees more with the patient monitor than the manual monitor. The Bland-Altman plot for Xiaomi HR against the manual monitor displayed a HR underestimation by 2.12 beats per minute and a limit of agreement between -15.07 and 10.83 with only one outlier over a 95% confidence interval (Figure 4(d)). Similar to the case of Fitbit, Xiaomi recorded large limits of agreement when compared to the manual monitoring device than to the patient monitor. The Fitbit device used does not measure SpO2 data; however, the Xiaomi device does. The Bland-Altman plot for Xiaomi SpO2 data against the Philips MX450 patient monitor SpO2 data revealed a mean difference of 1.11% on day one, -0.78% on day two, and an overall of -0.33%. The plot also showed a narrow limit of agreement between -3.47 to 2.81, which is because all of the SpO2 readings recorded were between 95% and 100% (Figure 4(e)).

The outliers in the Bland-Altman plots (extreme peaks) observed in Figure 3 for the Xiaomi wearable device may be due to artefacts due to unexpected movements of the volunteer during the data collection process. The fact that these movements were not detected for the Fitbit wearable device may reflect differences in the ability of the two algorithms used in interpreting data from the sensors of the respective CWDs to detect and correct for anomalies in measurements.

The accuracy of the devices was measured by a metric known as the mean absolute percentage error (MAPE) as explained earlier. From the MAPE (Table 3), the accuracy of the devices with regard to HR and SpO2 data was calculated in percentages. Fitbit recorded a HR accuracy measurement of 97.16% on day one, 96.6% on day two, and overall HR accuracy of 96.87% when compared to the patient monitor. Xiaomi recorded HR accuracy of 93.85% on day one, 94.61% on day two, and overall accuracy of 94.24% when compared to the patient monitor. Xiaomi again recorded SpO2 accuracy measurement when compared to the patient monitor as follows: 98.08% on day one, 99.01% on day two, and an overall accuracy of 98.79%. When compared to the manual monitor, Fitbit and Xiaomi recorded accuracies of 96.64% and 93.21%, respectively, for HR measurement.

The results from the correlation analysis using the Mann–Whitney *U* test demonstrated that we fail to reject the null hypothesis that there exists a significant relationship between the Philips MX450 patient monitor and the Xiaomi device in terms of heart rate measurement since the *p* value in this case (*p* value = 0.192666) is greater than 0.05 as expected (Table 4). In contrast, results obtained for Fitbit against the Philips MX450 patient monitor provides evidence that supports the alternative hypothesis (H_1_) since the *p* value (0.030022) generated was less than 0.05. Also, there is enough evidence to reject the null hypothesis that “there exists a significant relationship between the Manual Monitor and the wearable devices in terms of heart rate measurement” since the reported *p* values are less than 0.05. It is worth noting that the data collected from the manual monitor is instantaneous while that collected from the wearable devices is an average over a minute.

### 3.2. Web Application Development

**Figure 5:**
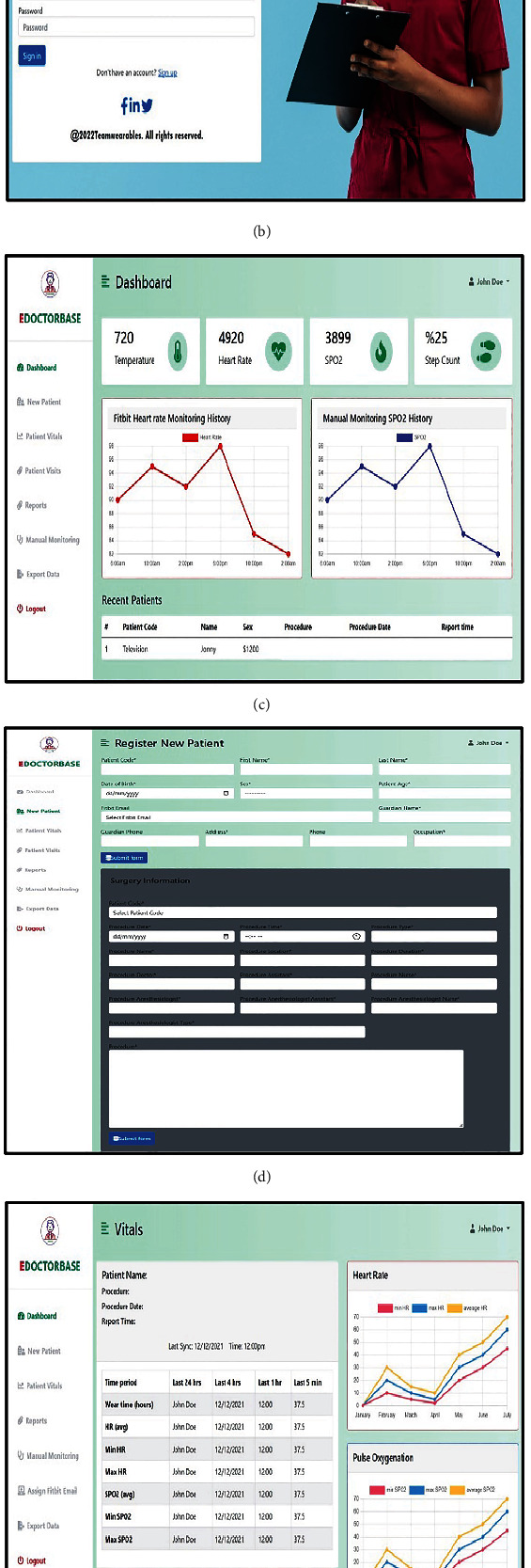
Pages of web application showing the main features and functions which include (a) user sign-up page, (b) user sign-in page, (c) individual user dashboard, (d) new patient registration page, (e) individual patient vital sign flowsheet with wearable device data shown in tabular and graphical form, and (f) vital sign upload page from manual measurements.

The system was designed with the following pages to achieve its functionality (Figure 5).

*Sign Up.* The user is required to provide the following credentials: first name, last name, username, designation, email, and password to sign up. A verification link is sent to the email provided earlier, and the user is required to click on the link to have access to the system.

*Sign In*. The user is required to provide a username and password as credentials to sign in to the system. When the email and password provided are valid, the system allows the user to have access to the system.

*Register New Patient*. The user can register a patient if it is the patient's first time visiting the hospital. The patient is given a unique outpatient department (OPD) number, which is used for identification. This number is used to identify each patient any time he or she visits the hospital. Additionally, the user is able to save the patient's surgical history in the system's database. This helps to maintain the medical history of the patient.

*Upload Patient Vitals*. Vital signs are taken manually in the hospital every four (4) hours and written in a document for safekeeping and records. The user is able to upload patient vitals into the system's database, which is a more secure way of storage. Also, the vital sign data can be retrieved as a CSV file whenever it is needed.

*View Patient Vitals*. Patient HR obtained from Fitbit web API is displayed here as a line chart. SpO2 data is also displayed as a line chart. The chart displays the minimum, average, and maximum HR and SpO2 trends. A table included also shows minimum, average, and maximum HR and SpO2 for the last 24 hours, 4 hours, 1 hour, and 5 minutes, respectively.

*Receive Alerts for Abnormal Vitals*. The table included in the vital visualization page alerts the user of abnormal HR and SpO2 data by changing the text color to red when vitals are high and blue when vitals are low. However, the text color remains black when vitals are normal according to the standard range for HR and SpO2.

*Export Data*. Users are able to export all patient information as a CSV file. In addition, they are able to export all patient surgical information, CWD data, and manual monitoring data, as CSV files. Users are also able to import data into the system's database.

Dashboard: Users can view recent patient information such as HR, SpO2, temperature, and step count.

## 4. Discussion

This project sought to assess the feasibility of using CWDs to monitor SpO2 and HR of patients in a quaternary care Ghanaian hospital. HR and SpO2 of a healthy male volunteer were successfully monitored in a hospital setting using Fitbit Versa 2 and Xiaomi Smart Band 6, showing high concordance between measures from these CWDs with the clinical gold standard. The CWD data were simultaneously integrated with the web application designed by the project team. Simulation was achieved by successfully connecting the CWDs wirelessly to the web application to display the vital signs measured in a concise and easily understandable format. These results demonstrate the feasibility of a monitoring system using CWDs with a web application that could be used in LMICs in the absence of the gold standard bedside monitor.

Our findings demonstrate the potential utility of CWDs in resource-limited areas throughout the world. CWDs may be used to supplement the scarce bedside patient monitors available and alleviate the heavy burden of understaffing in low-resource LMIC hospitals. With the virtual platform, use of such devices may also aid clinicians in viewing and following trends in patient vital signs, further improving the quality and timeliness of clinical decision-making. Several clinical conditions (e.g., trauma from car accidents, which is highly prevalent in these countries) could benefit immensely from having access to this technology. Trauma patients often suffer from internal hemorrhage which could result in the patients' death. Continuous monitoring of HR and SpO2 is vital to detecting such changes. Thus, using the CWDs could potentially save thousands of lives.

Recognizing that the current CWD measures are not ideal is important before implementing them in clinical settings. CWDs performed significantly better than manual monitoring and had high concordance with the gold standard, which is expected. However, despite the high concordance between measures from these CWDs with the clinical gold standard, differences in accuracy were still observed. Using the Bland-Altman analysis, it was determined that the Fitbit overestimated HR by less than 1 beat per minute on both days of the study, while the Xiaomi underestimated the HR by less than 1 beat per minute. SpO2 data analysis using the same method showed an overall underestimation of SpO2 by the Xiaomi of -0.33% on both days of the study. While the overall overestimations and underestimations were generally small for HR and SpO2, it is important to further explore the clinical implications of these differences in various patient populations, especially as the statistical test for concordance (Mann–Whitney *U*) revealed some differences between the CWDs and the gold standards for HR measurement.

Previous studies obtained larger error estimations and limits of agreements for Fitbit HR which is less accurate compared to the values obtained in this study [34, 35]. Another study in the literature reported that an error margin of less than ±10% has been widely accepted by organizations for wearable devices used for estimating HR [36], a margin within which our analysis shows the CWDs studied fall. Although many studies have been conducted on Fitbit, no study has been reported on the use of Xiaomi for vital sign monitoring. Further, this is the first known study in a low-resource LMIC to assess the concordance between CWD-derived vital signs and the gold standard vital signs obtained from a bedside monitor, while simultaneously interfacing the results with a centralized platform storing and displaying the vital signs for clinical use.

The Fitbit and the Xiaomi band were selected based on their robust design and wide use. While our findings have shown that both devices are fairly accurate compared to the gold standard, there may be advantages to one CWD over the other, depending on the patient population being monitored. However, all of these devices are continuously improving their sensing capabilities. The availability of other vital signs such as respiratory rate and body temperature that are measured by these devices will increase, ultimately furthering the case for the use of CWDs in low-resource hospitals.

This study has limitations. First, the study was conducted on one healthy individual over a short period of time. Data were not collected on a typical busy clinical ward with multiple patients. Thus, the results may overestimate the accuracy of the CWDs. Second, the interfacing of CWD data with the web application was not performed in real time. While we did demonstrate the feasibility of this interface, further testing and modeling are necessary to demonstrate the feasibility and utility of providing real-time vital sign data to clinicians in a clinical setting. Furthermore, although the web application was successfully launched on Heroku's cloud server and made publicly available, there was a time out error during the testing phase. This issue simply indicates that the internal server took longer to load the web application's resources. This could possibly be due to the web application's contact with the Postgres database server. Finally, no work was done on validating or mitigating against reported inaccuracies in SpO2 measurements taken from dark-skinned patients. Clearly, the system can benefit from further optimization. Replicating our findings using actual patients in various clinical settings and over longer periods of monitoring time is needed to further validate and certify the use of CWDs in hospitals in low-resource settings.

## 5. Conclusions

The two wearable devices used in this study (Fitbit Versa 2 and Xiaomi Mi Smart Band 6) were shown to be accurate in measuring oxygen saturation and heart rate and were easily interfaced into the web application. This concordance study will aid in the development, validation, and certification of these devices to be used alongside the fully functional web application developed by our team, both in Ghana and other low-resource low- and middle-income countries.

## Figures and Tables

**Table 1:** Spearman's correlation coefficient results for heart rate for Fitbit and Xiaomi against the continuous bedside monitor and manual monitor.

Device	Philips MX450 patient monitor	Manual monitor
Fitbit	0.749730	0.841943
Xiaomi	0.607200	0.422363

**Table 2:** Results from the Bland-Altman plot.

Devices	Mean difference	Limits of agreement	Outliers
Fitbit heart rate vs. patient monitor	0.77	-3.78 to 5.33	6
Fitbit heart rate vs. manual monitor	-1.56	-7.23 to 4.11	3
Xiaomi heart rate vs. patient monitor	-0.86	-10.56 to 8.82	15
Xiaomi heart rate vs. manual monitor	-2.12	-15.07 to 10.83	1
Xiaomi SpO2 vs. patient monitor	-0.33	-3.47 to 2.81	4

**Table 3:** Results from mean absolute percentage error (MAPE) calculations.

Device	Philips MX450 patient monitor	Manual monitoring device
Fitbit heart rate day 1	2.84%	
Fitbit heart rate day 2	3.40%	
Overall Fitbit heart rate	3.13%	3.63%
Xiaomi heart rate day 1	6.15%	
Xiaomi heart rate day 2	5.39%	
Overall Xiaomi heart rate	5.76%	6.79%
Xiaomi SpO2 day 1	1.92%	
Xiaomi SpO2 day 2	0.99%	
Overall Xiaomi SpO2	1.21%	

**Table 4:** Mann–Whitney *U* test results for Fitbit and Xiaomi against the continuous bedside monitor and manual monitor for heart rate measurement.

Device	Philips MX450 patient monitor		Manual monitoring device	
	Statistic	*p* value	Statistic	*p* value
Fitbit	31304.5	0.030022	5117	0.001564
Xiaomi	26147.5	0.192666	5111	0.001524

## Data Availability

The data used to support the findings of this study are available from the corresponding author upon request.
